# Endoluminal sclerosis with diode laser in the treatment of orofacial venous malformations

**DOI:** 10.4317/medoral.18528

**Published:** 2013-03-25

**Authors:** Juan C. Álvarez-Camino, Antonio J. España-Tost, Cosme Gay-Escoda

**Affiliations:** 1 DDS. Resident of the Master of Oral Surgery and Implantology. University of Barcelona Dental School; 2 DDS, MD,PhD. Associate Professor of Oral Surgery. School of Dentistry of the University of Barcelona. Investigator of the IDIBELL Institute. Director of the Master of Dentistry Laser. University of Barcelona. Coordinator of the European Master Degree in Oral Laser Applications (EMDOLA); 3 MD, DDS, PhD. Chairman and Professor of Oral and Maxillofacial Surgery. Director of the Master of Oral Surgery and Implantology. School of Dentistry of the University of Barcelona. Coordinator/Researcher of the IDIBELL Institute. Head of the Oral and Maxillofacial Surgery Department of the Teknon Medical Center, Barcelona (Spain)

## Abstract

Introduction: The appearance of vascular anomalies in the orofacial area is a common condition, which represents about 50% of these malformations. Traditional treatment approach, such as surgery and chemical sclerosis has been given way to a few less-invasive options, as the use of the 810nm diode laser to induce the sclerosis of the venous malformation by intralesional photocoagulation. Objectives: The objective of this study was to determine the efficacy of the diode laser in the intralesional treatment of the orofacial venous malformations (OFVM), describing the recommended surgical approach, as well as to report the main associated complications. Patients and Methods: 10 cases of OFVM, diagnosed and treated at the Oral Surgery Department of the Dental Clinic of the University of Barcelona, between January, 2009 and April, 2011. Every case was treated under local anesthesia, performing at least one intralesional session of diode laser, applying an 1W active optic fiber, in continuous mode, inserted into the interior of the lesion through an intramuscular needle, from the deepest portion to the surface of the lesion. Postoperative medication was indicated and follow-up visits were perform during a period of at least 6 months. Results: Of a total of 10 cases of OFVM, mean age of 25.4 years, 8 required just a single session with intralesional laser diode, before the clinical verification of a total reduction of size of the lesion. In 2 of these cases, were needed at least 2 sessions of intralesional photocoagulation to reach a satisfactory cosmetic result. No complications of any kind occurred. After a follow-up period of at least 6 months only a case of recurrence was described. Discussion and Conclusions: The advantages associated to the use of non-invasive techniques in the treatment of OVM, along with the success rate and low number of relapses, shows the use of the diode laser as a therapy to be considered in the treatment of these lesions. A higher case mix would be essential for definitive conclusions.

** Key words:**Diode laser, hemangioma, orofacial venous malformation.

## Introduction

Treatment of patients with vascular anomalies in the craniofacial area usually represents a major clinical challenge because 60% of these cases occur in young patients (1). Interdisciplinary diagnosis and treatment becomes of real importance in these cases. One of the main problems of the clinician at the time he had to deal with one of these cases, was the need of get a satisfactory classification of the lesion. In 1982, Mulliken and Glowacki (2) proposed a classification of vascular anomalies based on their clinical, histological and histochemical study. The classification of Mulliken and Glowacki groups the vascular anomalies into two major groups. The first group includes those tumors associated with a process of endothelial cell proliferation, which appears in childhood, with proliferative changes, followed by an involutional process of unknown etiology, called hemangiomas, and a second group consisting of those vascular malformations (VM) that appears as a result of the altered development and formation of blood vessels, present at the birth , normal endothelial mitotic activity, which grows with the individual and with no presence of an involutional process (2). These malformations can show any combination of capillary, venous, arterial or lymphatic components, presence or absence of fistula. Moreover, lesions can be differentiated according to the vascular flow, either low or high flow. Approximately 50% of these vascular lesions are located at the head and neck region (3).

Different methods of treatment of vascular lesions has been described: surgery, steroid therapy, embolization, cryosurgery, electrodesiccation, among others (4). The use of intralesional injections of sclerosing agents is also showed as an effective method in the treatment of VM. During the 90’s, the use of high power lasers in the treatment of vascular abnormalities is introduced, with the use of neodymium: yttrium aluminum garnet contaminated with neodymium (Nd: YAG) laser, which proved to be an effective treatment tool, although with limitations (4). Photocoagulation using laser Nd: YAG as a method of treatment of vascular lesions is based on the high capacity of absorption of light by hemoglobin, which in conjunction with the heat released by the absorption of laser light during the penetration of the tissues, produces a selective coagulation of the affected blood vessels.

The diode laser (GaAs, GaAlAs) is presented as an efficient, compact and portable surgical equipment, and represents a minor financial investment compared with other lasers equipments for surgical use. Diode lasers have a wavelength of between 805 to 980 nm. Can be used in both continuous and pulsed mode, as well as in contact or non-contact mode with the tissues, depending on the clinical indication. Applications in the non-contact mode are performed using different fibers or by focalization the hand-piece, as in the case of the coagulation of superficial lesions or the excision of soft tissue lesions (5).

Sclerosis photocoagulation by diode laser has been used extensively in the treatment of VM in areas such as dermatology and ophthalmology. Coagulation properties of the diode laser are particularly effective and beneficial in the treatment of VM (3). The diode laser light turns into heat because of its absorption by the hemoglobin. The penetration depth of the 810 nm wavelength appears to be lower than the one in the Nd:YAG, allowing it use in an effective way in the coagulation of superficial and intersti-tial lesions (5). Histological studies on vein walls in which a diode laser session was applied revealed that a coagulative necrosis took place, showing tissue distortion, not at the inner lining of the vessel only, but in outer layers of the vascular wall as well (5).

The objectives of this study are, using the report of cases treated in our department, to determine the efficacy of diode laser (? 810nm) in the intralesional treatment of orofacial vascular malformations (OFVM), describe the surgical technique used, and report major complications.

## Patient and Methods

A retrospective observational study of patients affected by OFVM was performed. These patients, who attended to the Oral Surgery Department of the Dental Clinic of the University of Barcelona, were diagnosed following the correspondent clinical criteria and were treated by using the intralesional sclerosis with diode laser during the period between January, 2009 and April, 2011. All cases were identified through clinical diagnosis. No complementary diagnostic tests were run, except in those cases where the size of the lesion was significant enough to suspect a deep affectation of the surrounding tissues, and to discard the existence of intraosseous shunts, when the injury was settled in the vicinity of cortical bone.

OFVM of all the patients were treated using a 810 nm wavelength gallium arsenide (GaAs) diode laser unit (Biolase ® LaserSmile ™, Biolase Technology, Inc., Irvine, USA.), using a 300?m optical fiber, preceded by perilesional anesthetic. All patients were treated in ambulatory settings, following the security protocol related to the use of a type IV laser.

The information was collected by a single observer; age, gender, size and localization of the lesion and postoperative follow up data were collected.

The data was processed using the Statistical Package for Social Sciences version 12.0 (SPSS, SPSS Inc., Chicago, Ill, USA, licensed to the University of Barcelona).

## Results

A total of 10 patients, mean age of 25.4 years, age range from 19 to 39, with a predominance of female patients (7:3) was re-ported. 80% (n = 8) of these patients presented OFVM lesions with a diameter of 3 cm (mean= 3.1 cm) or less. The upper lip (50%) was the most frequent location, followed by the dorsum of tongue (20%) ( Table 1).

**Table 1:**
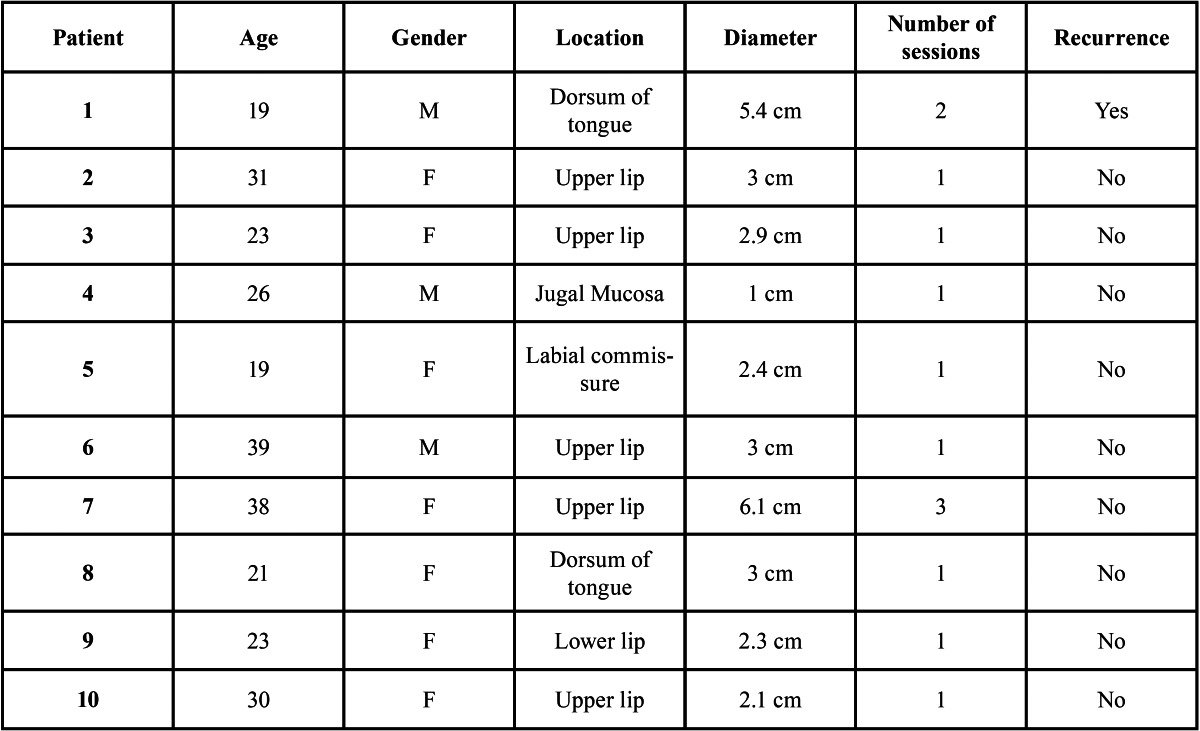
Clinical data of the OFVM included in the study.

The surgical technique begins with a perilesional infiltration of 4% articaine hydrochloride with epinephrine 1:100000, followed by the puncture of the lesion with a 20G angiocatheter (Abbocath, Abbott Laboratories, Illinois, USA) as initial approach, followed by the introduction of the previously activated optical fiber, via the catheter lumen until the active tip is positioned within the OFVM , keeping it accessible using the lighting guide of the laser unit. The laser is radially applied in the deeper areas of the lesion, while the laser fiber is progressively moved upward to the entrance area of the fiber. Subsequently, the optical fiber is removed and postoperative instructions are given; analgesic medication (paracetamol 1 g, one tablet every 8 hours for 4 days) and topical 0.2% chlorhexidine bioadhesive gel (2 times a day, every 12 hours for 15 days) is recommended. Follow-up visits are performed at 7, 30, 180 and 365 days.

80% of the treated OFVM required a single session of intralesional endoluminal treatment to completely remove the lesion, this was confirmed during the follow-up visits. Lesions with a diameter less than or equal to 3 cm required a single session. It was found that, in a case of OFVM (10%) located in the upper lip, diameter of 6.1 cm, although there was a significant reduction in the lesion size, there was not a complete involution of the lesion after 7 and 30 days postoperative evaluations, so it was decided that a second session with intralesional diode laser had to be performed. After a 30 days follow-up visit, an incomplete involution of the lesion was observed, so it was decided to perform a third session. During a follow up visit after the latter approach, it was found an important involution of the lesion, although the aesthetic commitment remained. The recurrence of the lesion has been described In one case (10%), after the 180 days follow-up visit. The lesion, located on the dorsal side of the tongue, presented an initial total involution observed after the first follow-up visits. After a second treatment session, and 7, 30 and 180 days follow-up visits, the complete regression of the lesion was observed.

## Discussion

Back in 1996, the International Society for the Study of Vascular Anomalies adopted the classification proposed by Mulliken and Glowacki, which classifies the vascular lesions in tumors (hemangiomas, etc.) and malformations (capillary, venous, arteriovenous, lymphatic, combined) (2). Hemangiomas, with its characteristic endothelial structure, tend to an after birth development, with an increasing tendency to grow during the first year of life, followed by a slow regression, presenting a complete evolution at 4 or 5 years in 50% of cases (1,4). Due to this fact, a passive attitude is recommended, performing regular follow-ups. Vascular malformations, on the other hand, are usually diagnosed at birth, grow with the individual, and show no tendency to regress on their own(1). Most authors report a relatively high incidence of OFVM (3). 50% of all benign lesions appear on the head and neck area(4), causing discomfort to the patient by its functional and aesthetic impact.

The diagnosis of venous malformation (VM), as well as of vascular tumors is usually based on their clinical features. High risk of bleeding during is the biopsy plus the benignity of the lesions, advise against conducting a histopathology study, despite being histologically different entities. However, the different treatment modalities described by the authors are similar for both entities.

Different therapeutic options are used in the treatment of benign vascular lesions. The choice will depend on the location, depth and the progression of the lesion. No treatment is necessary in a few cases; in the other hand, the lesion could be associated with serious sequelae such as growth, bleeding or cause critical airway obstruction, which would require immediate surgery (6).

In the case of hemangioma, periodic controls are included as a treatment option. This is because the lesion invariably regresses, and in some cases may remit completely, but frequent controls are recommended to evaluate the evolution of the lesion. In the case of small VM control may be an option, although there is controversy about the need of treatment (7). Surgical resection is complicated, and is associated with an increased risk of bleeding, difficulty of anatomical dissection, scarring sequelae, deformity and a high rate of recurrence (3). Cryosurgery can be a treatment option for early stages of some small, superficial vascular lesions, but carries a risk of atrophy, hypopigmentation and scarring in the skin of treated area (8).

One of the most commonly employed treatments for VM consists of intralesional injection of substances such as absolute ethanol and other sclerosing agents such as sodium morrhuate polidocanol (9). The mechanism of action is based on the damage of the endothelium, with a consequent inflammation process and the subsequent associated fibrosis. Despite this, and though is a highly protocolized technique, with a 70-90% success rate when performed by an experienced team, sequelae and complications have been described, such as cutaneous or mucosal necrosis, and peripheral nerve injury (3).

The use of different types of lasers in the treatment of vascular lesions was discussed in early studies such the one of Apfelger and Maser (10), that suggested that intralesional therapy using the Nd:YAG laser can be an effective treatment the MV. Initially, the Nd:YAG laser was used in the treatment of hemangiomas and VM by irradiating the surface of the lesion, in continuous mode to get the sclerosis of the lesion without puncturing; however, this technique is associated to necrosis of the irradiated area, due to the high capacity of tissue penetration presented in this type of laser, therefore, this technique requires the continuous local application of cold to avoid necrosis (5).

The diode laser has a selective performance over vascular tissues. It works in a 810-980 nm wavelength range, exhibiting a selective absorption by hemoglobin, with a tissue penetration as low as 0.3 mm, due to its high affinity for water, which forms 80% of the soft tissues (11,12). The intralesional fiber of the diode laser seeks to cause the endothelial injury of the affected vessel by both the thermal action caused by laser light, and the secondary thermal effect caused by the bubbles associated with the absorption of light energy by hemoglobin (13).

Different authors, like Angiero et al. (7), Lapidoth et al. (14), Genovese et al. (15), Puche-Torres et al. (3), perform the endoluminal sclerosis technique for both MV or hemangiomas at the orofacial area. Lapidoth et al. (14) even combines intralesional use of diode laser with radiofrequency, with a success rate of 90%, with few postoperative complications. Overally speaking, the success rate of treatment of MV with intralesional laser diode is positive, according to the published casuistry, especially in small superficial lesions. A success rate of 95.24% for one or two sessions of treatment is referred by Puche-Torres et al. (3).

Different investigators also report the recurrence of the VM after treatment with endoluminal sclerosis using the diode laser, although the incidence rate is quite low. Puche-Torres et al. (3) reported a recurrence rate of 4.76%. In our study, a single case of recurrence is reported.

The highest rate of complications is associated with an inadequate transfer of energy to the surrounding tissues, as in any technique in which laser is used. Extreme caution should be taken with mucous membranes and skin next to the OFVM. Injury can also occur in structures near the VM, such as nerves or excretory ducts. This circumstance should be taken into account during the treatment planning, and alternative methods, such as cryosurgery should be considered (7). To avoid these complications, an adequate safety margin should be keep, relative to the size of the lesion, trying not to keep the laser fiber too long at the same place, moving slowly within the lesion, observing the decrease in size and bleaching of the lesion.

Other lasers can achieve better success rates, because of its better absorption by hemoglobin. These include the argon laser and Nd: YAG KTP. Both emit a green light that matches the peak of maximum absorption of the hemoglobin.

## Conclusions

Treatment of OFVM by endoluminal diode laser is a practical technique, fast, simple, minimally invasive, performed under local anesthesia in an outpatient setting, with a low complication rate, with an excellent postoperative results, whenever an experienced and trained clinician is on the lead.
